# Infection Control in Dental Practice During the COVID-19 Pandemic

**DOI:** 10.3390/ijerph17134769

**Published:** 2020-07-02

**Authors:** Alessandra Amato, Mario Caggiano, Massimo Amato, Giuseppina Moccia, Mario Capunzo, Francesco De Caro

**Affiliations:** Department of Medicine, Surgery and Dentistry, Scuola Medica Salernitana, University of Salerno, 84126 Salerno, Italy; mamato@unisa.it (M.A.); gmoccia@unisa.it (G.M.); mcapunzo@unisa.it (M.C.); fdecaro@unisa.it (F.D.C.)

**Keywords:** COVID-19, dentistry, SARS-CoV-2, prevention procedures, guidelines, coronavirus infection, SARS virus, preventive dentistry, infection in dentistry, hand hygiene

## Abstract

COVID-19 is the disease supported by SARS-CoV-2 infection, which causes a severe form of pneumonia. Due to the pathophysiological characteristics of the COVID-19 syndrome, the particular transmissibility of SARS-CoV-2, and the high globalization of our era, the epidemic emergency from China has spread rapidly all over the world. Human-to-human transmission seems to occur mainly through close contact with symptomatic people affected by COVID-19, and the main way of contagion is via the inhalation of respiratory droplets, for example when patients talk, sneeze or cough. The ability of the virus to survive outside living organisms, in aerosol or on fomites has also been recognized. The dental practitioners are particularly exposed to a high risk of SARS-CoV-2 infection because they cannot always respect the interpersonal distance of more than a meter and are exposed to saliva, blood, and other body fluids during surgical procedures. Moreover, many dental surgeries can generate aerosol, and the risk of airborne infection is to be considered higher. The aim of this paper is to provide practical advice for dentists based on the recent literature, which may be useful in reducing the risk of spreading COVID-19 during clinical practice.

## 1. Introduction

During December 2019, a great number of patients suffering from a severe form of pneumonia were observed in Wuhan, the capital city of Hubei province, with a population of over 11 million, located in the center of the People’s Republic of China. The origin of the contagion was thereafter identified as the “Huanan Seafood Wholesale Market”, in which a wide variety of live wild animal species, including bats, snakes, and pangolins are sold [1]. This pathology was not attributable to any known etiological agent. On 9 January 2020, through the examination of the pharyngeal and respiratory swabs of hospitalized patients, a new coronavirus called SARS-CoV-2/human/Wuhan/X1/2019 was isolated. This virus will go down in history as the pathogen responsible for the pandemic spread declared by the WHO on 11 March 2020 [2].

The disease caused by the SARS-CoV-2 virus has been called, by the World Health Organization (WHO), COVID-19, an acronym derived from the terms CO-rona VI-rus D-isease and the year of identification-19 [3]. The most commonly stated symptoms are asthenia, myalgias, nasal congestion, rhinitis, pharyngotympanic, and especially a dry cough and dyspnea with fever. Some patients could also have a sore throat or diarrhea. The symptoms are mild in 80% of the patients and more severe in 15% of the cases so as to require hospitalization. In the last 5% of patients, the onset of severe dyspnea leads to direct access to an intensive care unit [4]. The data on human-to-human transmission and surface stability of SARS-Cov-2 are currently not totally clear. For these reasons, the same containment strategies for previous coronaviruses have been adopted.

Human-to-human transmission seems to occur mainly through close contact with symptomatic people affected by COVID-19, and the main way of contagion is respiratory droplets when patients sneeze or cough [5]. Although the virus is more contagious when the patient is symptomatic, a growing body of evidence suggests the possibility of human-to-human transmission even in patients with mild or absent symptoms [6]. The possibility that the virus can survive outside living organisms, in aerosol or on inanimate materials has also been recognized. A study published in the New England Journal of Medicine found that SARS-CoV-2 remained viable in aerosols for up to 3 h with a half-life of 1.5 h [7]. The virus can survive longer on stainless steel and plastic with an average half-life of approximately 5.6 h and 6.8 h, respectively, and the viable virus was detected up to 72 h after application on these surfaces. Because of the pathophysiological characteristics of the COVID-19 syndrome, the particular transmissibility of SARS-CoV-2, and the high globalization of our era, the epidemic emergency from China has spread rapidly all over the world.

In Italy, the first local cases were officially detected on 21 February 2020, with the identification of three clusters located in Veneto, Emilia Romagna, and Lombardy. Since then, the Italian government has ordered restrictive measures and social distancing that were gradually extended to the whole country.

With the aim of containing the spread of COVID-19, many medical clinics including dental surgeries and clinics have drastically reduced patient access by limiting clinical activity only to urgent and non-delayed care. The dental practitioners are particularly exposed to a high risk of SARS-Cov-2 infection due to the inability to maintain an interpersonal distance of more than one meter and to the exposure of saliva, blood, and other body fluids during surgical procedures. Moreover, many dental procedures can generate aerosol [8].

The aim of this paper is to provide practical advice to dentists based on the current literature, which may be useful in reducing the risk of COVID-19 spread, especially during the phases following the acute epidemic period. The literature search was conducted by PubMed library for a recently published article on the clinical and epidemiological features of SARS-CoV-2, and on COVID-19 spread, using the following search terminologies: “SARS-CoV-2 or 2019-n-CoV” or “COVID-19”, “COVID-19 in Dentistry”, “COVID-19 Spread”, “Surface Stability of SARS-CoV-2”, “SARS-CoV-2 and aerosol”, “prevention COVID-19 spread”, “Airborne Infection in dentistry” ”PPE and COVID-19”, “PPE and Dentistry”, and “Air-Conditioning and COVID-19”.

The search criteria identified 64 potential papers, which were reduced to 55 after deleting articles in Chinese because of our lack of knowledge of the language. Abstracts were reviewed by A.M. and M.C. to identify those relevant to the aims of this paper, for which the full papers were then obtained. After additional hand searches of identified papers, 30 studies fulfilled the inclusion criteria for this article.

## 2. Patients Screening

During their daily practice, dentists use rotary instruments such as handpieces or ultrasonic scalers with water cooling systems and air-water syringes. These instruments create a visible spray which contains large particle droplets of water, saliva, blood, and microorganisms. This aerosol production is potentially dangerous and is very difficult to contain [9]. For these reasons, meticulous patient screening before entering the dental office/clinic is obligatory. Recognizing affected COVID-19 patients with poor symptoms through a telephone survey before they reach the dental office could be the best way to prevent spreading the disease inside the dental studio.

According to the work published by Chinese Researchers on The New England Journal of Medicine (NEJM), fever with body temperature above 37.5 degrees occurs in 88% of COVID-19-positive patients [10]. Although a minority of apyretic cases have been found, a telephone survey could be conducted through a questionnaire which considers the most frequent symptoms of SARS-CoV-2 infection, potentially dangerous epidemiological links, and the transmission time.

### An Example of the Survey Could Have the Following Questions

- Have you received a COVID-19 diagnosis in the past 30 days? If YES, have you been confirmed recovered after two negative swab tests for SARS-CoV-2?
- Have you had a fever higher than 37.3 degrees in the past 14 days, or have you had any symptoms of dry coughing, pharyngeal pain, nasal congestion, fatigue, headaches, myalgia, diarrhea, and digestive discomfort or general sickness?
- Have you lost your sense of taste or smell in the past 14 days?
- Have you been in contact with people who have tested positive for COVID-19 in the past 14 days?
- Have you traveled or have you been in contact with people from areas with a high epidemiological risk in the past 14 days?
- Have you recently attended events such as conferences, seminars, or gatherings where a large number of people were present in the last 14 days?

**Figure 1:**
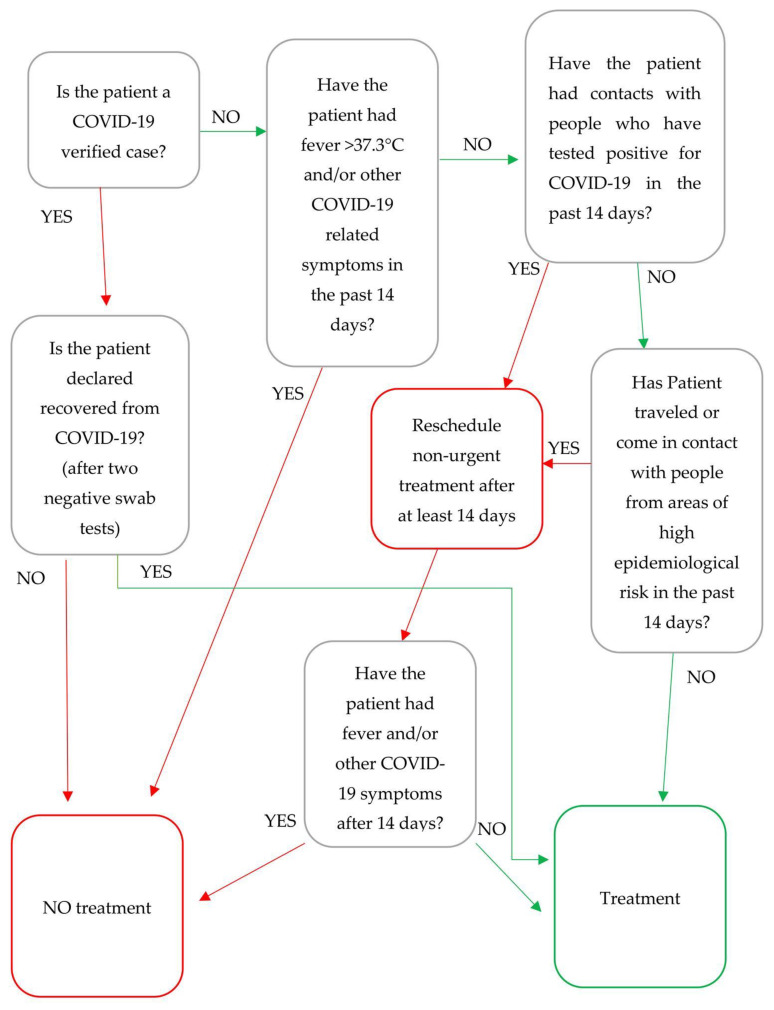
Patients flowchart screening for non-emergency dental care.

In the presence of fever detected in the last 14 days, positive epidemiological links, clinical signs, or symptoms related to COVID-19 (even with normal body temperature), it is preferable to reschedule non-urgent treatments after at least 14 days, recommending patients to contact their general practitioner for further investigation. It is also advisable to ask the patient to measure his/her body temperature every 8 h starting from the day before the appointment and to postpone the appointment for at least 14 days if it goes over 37.3 °C (Figure 1).

In case of urgent treatment on a confirmed, possible, or suspected COVID-19 patient, it is better to treat the patient in a public hospital by appropriately trained and equipped healthcare personnel. In the dental office/clinic, it is obligatory to schedule the appointment to the end of the day (maybe in a negative pressure or well-aerated room) using a dental rubber dam, avoiding all aerosol-generating procedures and minimizing intervention time. All disposable items including patient and workers Personal Protective Equipment (PPE) must be disposed of in a separate bag and with the modality proposed in Section 8.

## 3. Patient’s Entry

Patients must always wear a surgical mask at the dental office/clinic and if/when possible, must be unaccompanied (no partners/relatives); personal items are to be avoided. Nannies and caregivers must be treated in the same way as patients and will have to remain in the waiting room for the entire duration of the patient’s treatment.

These rules must be given to the patient via a detailed recommendation provided at the time of the scheduled appointment. Upon entering, an operator equipped with gloves, a filtering facepiece particles 2 (FFP2) respirator, visor, and a protective gown will measure the patient’s body temperature using an infrared thermometer, avoiding any contact with the patient’s body surfaces. If the patient’s body temperature is over 37.3 °C, it is advisable to postpone the appointment, especially in case of non-urgent care.

**Figure 2:**
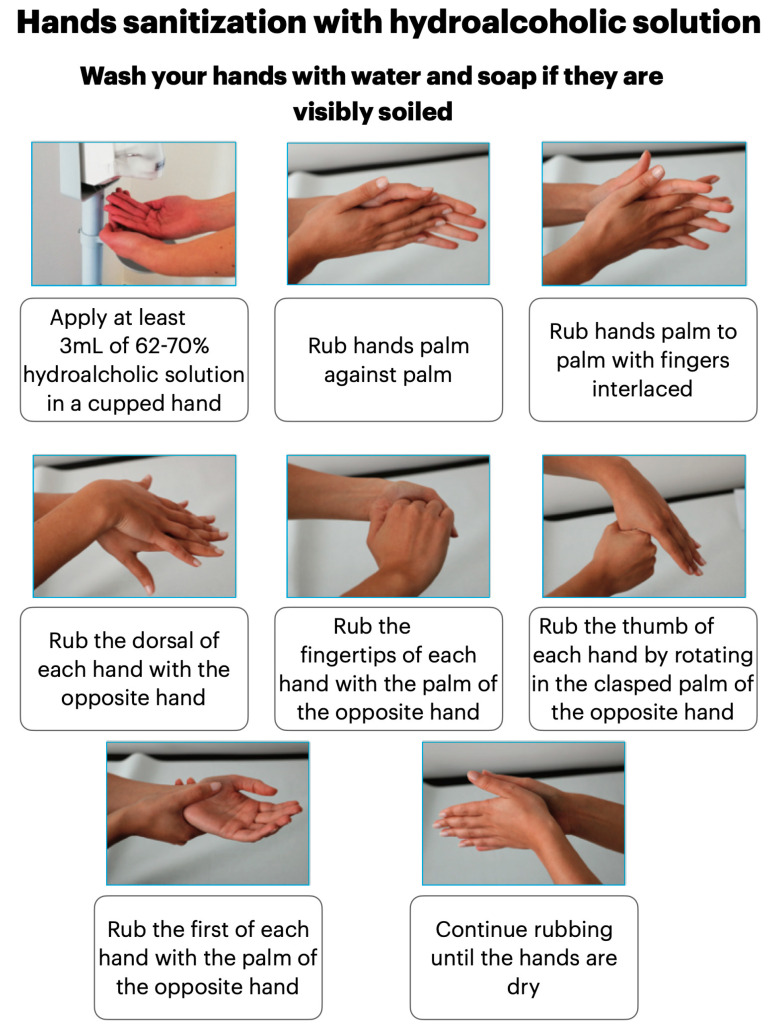
Hands sanitization with hydroalcoholic solution.

Then, the patient will be requested to leave any overcoats, bags, and backpacks in specifically organized boxes or spaces. He will be asked to throw the surgical mask in a special closed container and to sanitize his/her hands with a hydroalcoholic solution (Figure 2). At this point, the patient is equipped with goggles, disposable shoe covers, a gown, headgear, and a surgical mask, after which he/she must remain seated in the waiting room until he/she is called to enter the clinical area. Patients must wear the provided PPE until the end of the clinical procedure.

## 4. Appointment Planning and Waiting Room Organization

As SARS-CoV-2 is primarily transmitted among people through respiratory droplets, it is obligatory to schedule appointments in order to ensure that the number of patients in the waiting room allows the interpersonal distance of at least 2 m to be respected [11]. In order to avoid a large number of patients sharing the waiting room, the estimated time of any procedure should exceed at least 30 min. This extra time should be considered for the schedule organization.

Elderly patients or those with multiple chronic systemic diseases are considered more vulnerable in case of COVID-19 infection. For this reason, it is advisable to schedule their appointment at the beginning of the working day [12]. Ornaments, magazines, newspapers, and posters must be removed from the waiting room in order to improve cleaning and because SARS-CoV-2 could survive on paper and cardboard for up to 48 h [7].

If there is a desk at the reception, it is recommended to place plexiglass separators to protect the staff from droplets. The receptionist must wear a surgical mask and disposable gloves, which must be replaced after each patient. He/she is responsible for sanitizing all objects that come into contact with patients during administrative and payment procedures.

## 5. Access Modalities for Dental Office/Clinic Staff 

All dental office/clinic workers (dentists, dental hygienists, assistants, receptionists, etc.) must measure their body temperature daily, at least in the morning and in the evening. If the body temperature is higher than 37.0 °C, the operator must not go to work, and sanitary observation should be activated.

The dental workers must enter the dental office/clinic wearing a surgical mask, then immediately put on shoe covers, throw the mask in a special closed container, and clean their hands with a disinfectant hydroalcoholic solution (Figure 2) or with running water and soap, for at least 1 min.

In a personalized locker room, the staff must leave their clothing and other personal items in individual lockers and wear washable clothing and footwear. At the end of the procedure, it is essential for them to sanitize their hands again with hydroalcoholic solution (Figure 2). All dental office/clinic workers must maintain social distancing of at least 1.5 m among themselves and always wear a surgical mask. They must avoid staying in common eating and relax areas, etc. at the same time unless for strictly necessary reasons.

## 6. Personal Protective Equipment for Non-Sterile Procedures

As the patient prepares to enter the clinical areas, the operators prepare themselves with disposable personal protective equipment (PPE).

Since the initial Chinese observations, it already became clear that COVID-19 was able to be transmitted through direct or indirect contact of body fluids or droplets infected with ocular, oral, and nasal mucous membranes [13]. Consequently, the use of PPE as visors or protective glasses, fully covered disposable gowns, respirator/masks, gloves, and headgear caps are strongly recommended.

Operators must sanitize their hands before and during the PPE dressing procedures. According to the results of a Spanish study, frequent surgical washing with disinfectant soapy solutions could cause skin lesions and an alteration of the bacterial flora, allowing colonization by staphylococci and Gram-negative bacilli. Instead, hand cleansing with hydroalcoholic gel at a concentration level between 62% and 70%, seems to be tolerated well after frequent washing too. Episodes of contact dermatitis have also been described much more frequently with the use of soapy solutions compared to alcoholic hand sanitizers [14].

**Figure 3:**
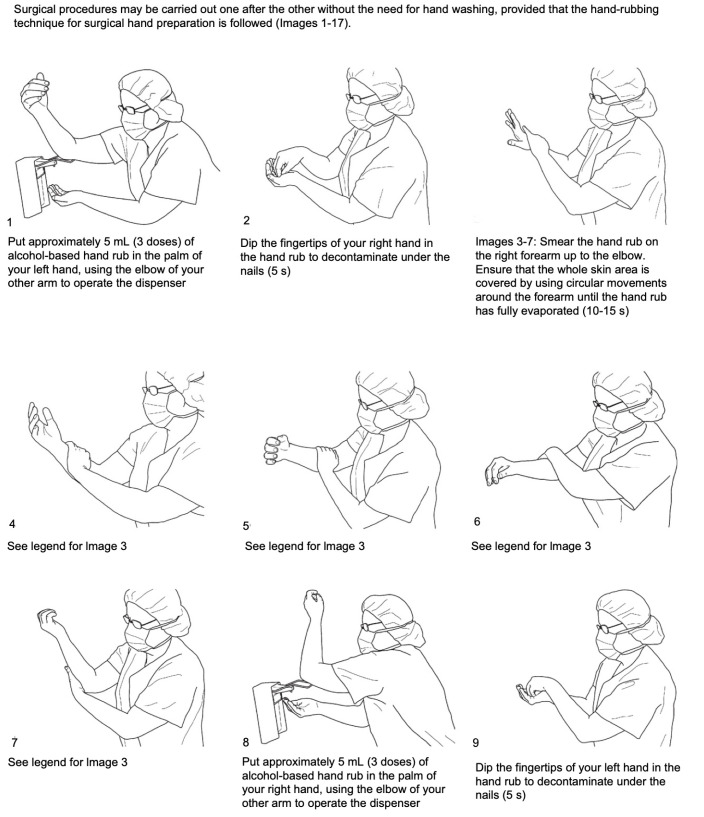

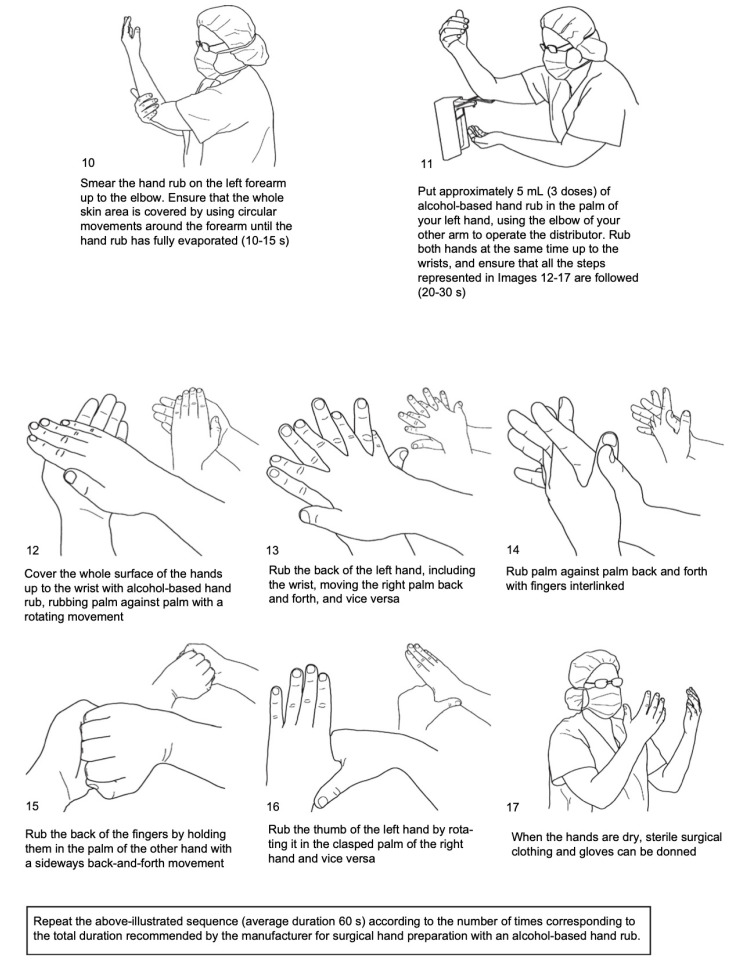
Hand-rubbing technique for surgical hand preparation. From WHO guidelines of hand hygiene in health care [15].

The WHO hand-rubbing technique for surgical procedures requires application on perfectly clean dry hands, of approximately 5 mL of alcohol-based gel, on the palm of the hand and rubbing the skin surfaces of both hands and forearms for about 60 s until the product is totally evaporated. The emollients and alcohol present in them could cause temporary pain or burning sensations if there are cuts or abrasions on the hands (Figure 3).

In case of contamination with blood and biological material, surgical hand washing under running water and soap is obligatory [16]. Therefore, we recommend sanitizing hands daily with alcoholic sanitizer solutions and limiting surgical hand washing only in cases of contamination.

### Operational Sequence for Wearing PPE

**Figure 4:**
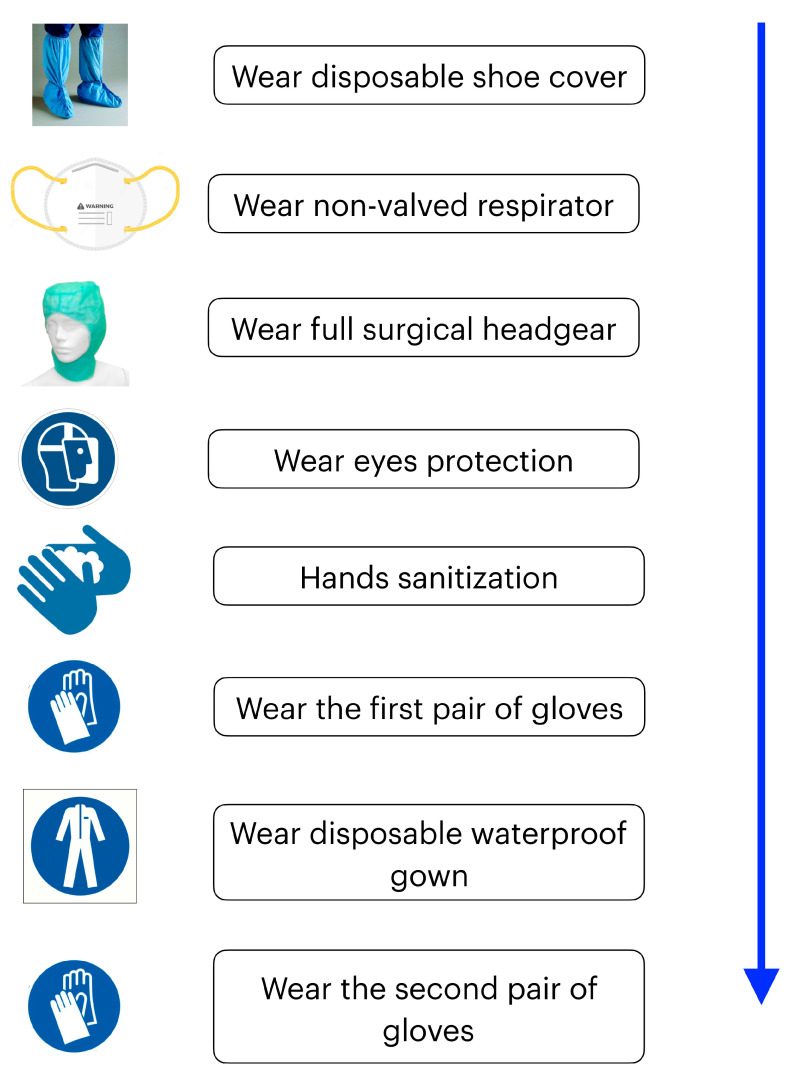
Operational sequence for putting on Personal Protective Equipment (PPE).

- Wear disposable shoe covers to protect washable studio rubber footwear.
- Wear a high filtered non-valved respirator (EU FFP2/ NIOSH N95 or EU FFP3/ NIOSH N99), ensuring its face fitting. Commonly used surgical masks do not provide complete protection against inhalation of airborne infectious agents of less than l µm [17]. The use of FFP3 respirator is mandatory in case of emergency treatment of a COVID-19 confirmed or suspected patient.
- Wear full surgical headgear, goggles, or visors to protect the eyes.
- Surgical hand sanitizing with a hydroalcoholic solution for 60 s (Figure 3) washing hands under running water at a minimum temperature of 37 °C and with disinfectant liquid soap solutions respecting action times. Care must be taken to carefully rub nails, fingers, palms, back of the hands, wrists, and parts of the forearms; then, rinse thoroughly under running water and dry with disposable wipes (dabbing). Alternatively, wash hands with a hydroalcoholic solution using the same skin fractionation methods for 60 s (Figure 3).
- Wear the first pair of latex or nitrile gloves (the latter are better for a lower surface porosity).
- Wear a disposable waterproof gown and a second pair of disposable gloves. (Figure 4)

## 7. Clinical Procedure

The patient, previously equipped with appropriate PPE (see Section 3), is seated on the dental chair unit which has been previously prepared with a removable barrier to protect its non-sterilizable parts. Patients must remove their surgical masks at the beginning of the clinical procedure and wear them again at the end.

A clinical valuation conducted by the Researcher of the Li Ka Shing Faculty of Medicine of the University of Hong Kong reported a consistent detection of SARS-Cov-2 in the saliva of 92% (11 of 12) of analyzed patients [18]. With the purpose of reducing the presence of the viruses in the saliva, some authors have suggested using a mouthwash with 1% hydrogen peroxide-based solutions by rinsing for at least 30 s [19]. However, although a strong sensitivity of the SARS-Cov-2 virus to oxidizing agents has been demonstrated in vitro, there are no tests which assess their real efficiency in vivo or which have studied the possible side effects deriving from the use of these substances as a mouthwash. 

According to the guidelines for the Diagnosis and Treatment of Novel Coronavirus Pneumonia (the 5th edition) released by the National Health Commission of the People’s Republic of China, oral rinses with mouthwashes containing chlorhexidine are not effective for SARS-CoV-2 [20]. 

During clinical procedures, the use of the rubber dam is strongly recommended to limit the spread of aerosols and potentially infected biological material. In a study published by Samaranayake, the use of rubber dams has been shown to significantly reduce airborne particles by 70% within 1 m of the operating range [21]. The use of high-speed handpieces without anti-retraction valves should be limited during the epidemic of COVID-19 due to the risk of virus and bacteria aspiration in the air and water tubes, which could potentially cause cross-infection for the contamination of the dental unit [22]. Air–water spry syringes must be used carefully and only when strictly necessary.

Some authors advise avoiding the application of intraoral X-ray techniques during the epidemic period from COVID-19 because they could trigger emetic reflexes and coughing, contributing to the formation of aerosols and the spread of infected biological enhancement material [19,23]. However, it should be considered that intraoral radiographic techniques emit a radiation dose that is 3 to 5 times lower than Panoramic and 40 times lower than the Cone Beam Computed Tomography (CBCT) [24]. Care must be taken to avoid improper use of radiographic techniques, subjecting the patient to excessive and unjustified exposure to ionizing radiation.

When intraoral imaging is required, sensors must be doubly covered to prevent perforation and must be correctly disinfected after use to avoid cross-contamination.

All dental and surgical procedures should be carefully carried out to prevent coughing and gag reflexes [20]. For example, the use of an intra-oral scanner compared to conventional “analogical” techniques is better when carrying out dental impression.

The treatment room door must be closed during interventions to avoid aerosol dispersion in other environments, especially if the air conditioning systems are switched on [1].

## 8. Removal of Personal Protective Equipment

At the end of the clinical intervention, it is obligatory for the operator to throw all disposable PPE (gloves, masks, protective gowns, headgear, shoes) inside special double-layered garbage bags, spraying them with a 0.5% hypochlorite solution, which will be sealed with a knot and temporarily stored in a closed container with a pedal opening. The discarded equipment will then be finally thrown out, respecting all the given directives of medical waste disposal. Following the test conducted by the Institute for Hygiene and Environmental Medicine of the University of Greifswald in Germany on the persistence of coronaviruses on inanimate surfaces and their inactivation with biocidal agent, the sanitizing of goggles and visors must be done by wiping them with 62–71% hydroalcoholic solutions or leaving them to soak for at least 1 min in a 1% hypochlorite or 0.5% hydrogen peroxide aqueous solution [25]. The same disposal procedure must also be applied to the PPE given to the patients.

### Effective Sequence for the Removal of PPE

**Figure 5:**
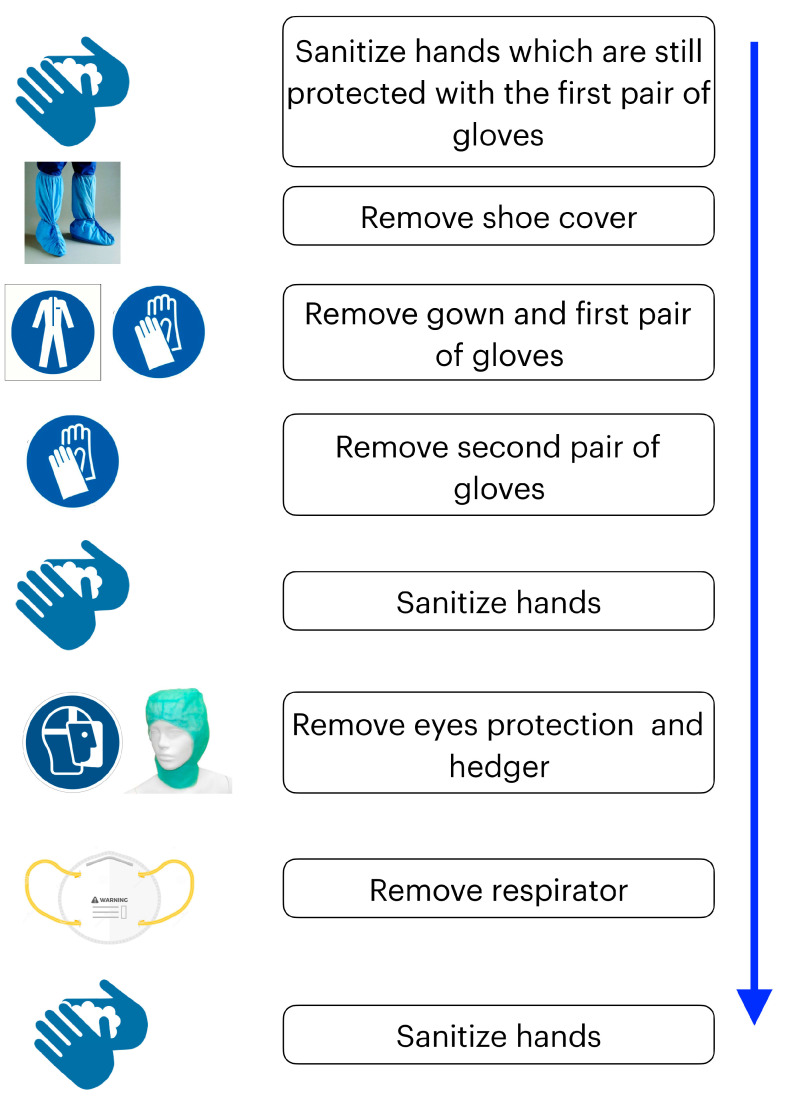
Effective sequence for the removal of PPE.

- Sanitize hands which are still protected with the first pair of gloves;
- Remove shoe covers and disposable gown;
- Remove second pair of gloves;
- Sanitize hands with a hydroalcoholic solution;
- Remove headgear, goggles, respirator;
- Sanitize hands and forearms with a hydroalcoholic solution;
- Recommended shower at the end of the working day (Figure 5).

## 9. Treatment Room Disinfection

At the end of the dental treatment, the patient must remove his/her disposable gown and other PPE, which must be carefully managed and disposed of in the same way as for the dental operators.

After removing the first pair of gloves, the dental assistant must collect all the contaminated tools, put them in appropriate containers in the disinfection and sterilization area. The common sterilization protocols adopted regularly by the dental offices/clinics are still effective for the prevention of COVID-19 cross-infection, which can be annihilated in the common autoclaves because it is not able to survive more than 30 min at temperatures above 56 °C [26]. Accurate sanitizing must be carried out on all surfaces of the dental unit, especially in the spittoon area, and must be extended to the dentist and assistant’s stool. It is best to spray and leave an aqueous solution of hypochlorite at 1% or alcohol at 70% for at least 1 min, passing from the cleanest to the dirtiest areas [25]. Wipe with disposable cloths, being careful not to go over previously treated surfaces.

Other measures may include cleaning the water lines of the dental unit before each use with a 0.5% hypochlorite solution, because residual water may be contaminated by viruses and bacteria. It has been proven that SARS-CoV-2 is able to remain suspended in aerosol for up to 3 h; for this reason, it is obligatory to carry out a complete air change in the clinical space after each intervention in order to reduce the risk of airborne infection, especially if high speed or ultrasound instruments have been used [7].

A continuous air exchange is possible through the use of air suction, filtration, and sanitary systems such as fixed devices with plasma cluster ion technology or UV lights and portable air cleaner with High-Efficiency Particulate Air (HEPA) filters or in dedicated negative pressure rooms [27]. The use of these devices is obligatory in rooms or dental offices/clinics without direct windows. Moreover, an additional high-volume evacuation device could help to reduce aerosol formation and droplet emissions during a dental procedure, particularly those which involve ultrasonic scaler use.

Windows with at least 2 square meter openings guarantee an efficient room air exchange in 10 min for a 20 square meter room; they must be opened after each patient [28].

Since SARS-CoV-2 is able to remain active for several hours on surfaces, it is obligatory to regularly disinfect everything that has been touched by patients or staff (door handles and taps, clean the waiting room seats, PC keyboards, mouse, drawers, filing cabinets). Sanitizing the floors and other surfaces can be carried out at the end of the working day with 1% solutions of hypochlorite.

Alternative procedures, such as ozone disinfectant technologies, could be considered to improve sanitizing because they combine a similar antiseptic ability to a liquid sanitizer with a better distribution on the surfaces [29]. It is still unclear if the use of air conditioning devices may be potentially dangerous for the spread of COVID-19. A Chinese study was conducted with the aim of clarifying a contagion experience that occurred inside a restaurant involving three families seated at different tables [30]. The distance between each table was about 1 m and all rooms in the restaurant had air conditioning. The authors conclude by suggesting that in this outbreak, droplet transmission was developed by air-conditioned ventilation and that the key factor for infection was the airflow direction.

Therefore, it is important to consider this finding when the use of ventilation or air conditioning systems is needed. However, this phenomenon could be used in the management of the aerosol produced after the dental treatment within the operating areas. For example by directing the air flow towards an open window could allow a faster air exchange and reduce the accumulation of potentially dangerous aerosols inside a closed environment.

When the use of air conditioning systems is needed, daily cleaning and sanitizing filters and components with hydroalcoholic solutions which come into contact with the air must be carried out. Detergents containing possible toxic substances which are dispersed in the air when air conditioners are on should be avoided. Systems which have HEPA or Ultra-Low Air Penetration (ULPA) filters are preferable. In the case of centralized air conditioning systems, maintenance must be carried out following the manufacturer’s instructions and recirculation must be excluded so as to avoid possible virus migration to other environments. However, an air exchange after each patient is recommended.

## 10. Conclusions

The pathophysiological characteristics of the COVID-19 syndrome, the particular transmissibility of SARS-CoV-2 make dentists and all dental workers highly exposed to a risk of infection. Efficient sanitizing procedures combined with the correct use of PPE can significantly reduce the probability of SARS-CoV-2 being transmitted during dental practice. Moreover, this virus is an invisible and extremely sneaky enemy that can be transmitted in several ways. Social distancing, correct behavioral rules, an adequate air exchange of all the dental office/clinic rooms, validated instrument sterilization, and surface sanitizing protocols, can reduce the risk of spreading SARS-CoV-2 but not to resetting it.

For these reasons, we strongly believe that a careful screening of each patient before entering the dental office/clinic is required. The possibility of recognizing affected COVID-19 patients with poor symptoms through a telephone survey may be the best way to prevent the spreading of the disease inside the dental office/clinic. With future knowledge, prevention protocols for the spread of COVID-19 may certainly undergo changes, perhaps with a simplification of the procedures when vaccines or rapid diagnostic tests are introduced to intercept even symptomatic or asymptomatic patients.

With this protocol, we hope to help the dental societies and authorities who are still working to publish complete and detailed official guidelines.
